# Correlation between sonographic follow-up of follicular growth, serum and salivary estradiol in women undergoing controlled ovarian stimulation (IVF/ICSI)

**Published:** 2018-12

**Authors:** AS Rottiers, L Dalewyn, S Somers, MM Alper, D Sakkas, J Gerris

**Affiliations:** Women’s Clinic, Department of Reproductive Medicine, Ghent University Hospital, 9000 Ghent, Belgium;; Boston IVF, 130 Second Avenue, Waltham Massachusetts 02451, United States.

**Keywords:** controlled ovarian stimulation, estradiol, follicle diameter, IVF, ICSI, salivary estradiol, transvaginal ultrasound

## Abstract

**Aim of the study:**

Investigation of the correlation between serum estradiol (E2), salivary E2 and sonographic measurements of follicles in women undergoing controlled ovarian stimulation (COS) for IVF/ICSI.

**Methods:**

This is a prospective study performed at the Department of Reproductive Medicine of Ghent University Hospital (Belgium) between November 2016 and January 2017 over a total of 40 patients. During routine COS, two-dimensional measurements of the follicles were performed using transvaginal ultrasound (TVUS) and E2 was measured in saliva and serum. A linear Mixed-Effects model (MIXED) was built, using SPSS Statistics 24.

**Results:**

Statistical analysis shows a strong linear correlation between serum and salivary E2. For every single unit increase in serum E2 (+ 1 ng/L) the estimated saliva E2 concentration is expected to increase with 0.011 pg/mL (95% CI [0.009 - 0.01]). Strong linear correlations between both saliva and serum E2 and follicular dimensions were also found. For every millimetre increase in follicle diameter the estimated serum E2 concentration is expected to increase with 8.32 ng/L (95% CI [7, 10-9, 54]). For every millimetre increase in follicle diameter the estimated saliva level of E2 is expected to increase with 0.11 pg/mL (95% CI [0.09 - 0.13]).

**Conclusions:**

A strong correlation between serum and salivary E2 concentrations was found. In addition, both are strongly correlated with the product of the number of follicles and their average diameter, measured by TVUS. More investigation needs to be done to find out if salivary E2 is an effective tool for monitoring IVF cycles.

## Introduction

Cycles of controlled ovarian stimulation for in vitro fertilisation (IVF)/intracytoplasmic sperm injection (ICSI) must be closely monitored. In general, this monitoring is performed using serial transvaginal ultrasound (TVUS) and measurements of serum estradiol (E2) (Kwan et al., 2014). One reason for monitoring is early prediction of ovarian hyperstimulation syndrome (OHSS), a potential life-threatening complication of ovarian stimulation with gonadotropin receptor hormone (GnRH)-agonists, which can lead to haemoconcentration, coagulopathy, pleural and pericardial effusion and hepatorenal failure (Devroey et al., 2011). Another reason for monitoring is the need to timely administer the injection of human chorionic gonadotropin (hCG) or agonist to initiate the final stages of oocyte maturation. When a certain number of follicles grow beyond a defined size (≥18 millimetre), the trigger to cause an ovulation is given (Kwan et al., 2014). Measurement of E2 levels in serum is used to support the decision of giving a trigger for ovulation. Moreover, it is known that the level of E2 can be used as a predictor for OHSS (Aboulghar, 2003). D’Angelo et al. (2004) showed that a level of E2 in serum of > 3500 pg/mL on day 11 of ovarian stimulation in an ART-cycle predicts the occurrence of OHSS with a sensitivity and specificity of 85%.

Although monitoring using TVUS and serum E2 is used as the clinical standard (Kwan et al., 2014), it entails some disadvantages. Monitoring is time-intensive and relatively expensive. As it is difficult to predict when patients need to come for monitoring due to the variability of individual responses on stimulation, it causes stress for patients as well as for care-providers. In rural areas, patients have to travel great distances to the nearest hospital (Gerris and De Sutter, 2010). Moreover, a venepuncture is an invasive procedure. The aim of this study was to examine whether it was possible to simplify monitoring of cycles for IVF and ICSI by using saliva sampling. Since patients can perform this at home, it could be a time- and cost-saving alternative for phlebotomy.

Saliva is an excellent medium because it is a natural ultrafiltrate of blood, it reflects the biologically active (free) fraction of steroids in the bloodstream (Kells et al., 2009). Steroid hormones are not bound by carrier proteins. 1 to 10% of the steroids in blood, leak into saliva from plasma. Albumin and sex hormone-binding globulin (SHBG) do not allow the bound fraction of the hormones to get into saliva due to their molecular weights (Celec et al., 2009; Kells et al., 2009).

We investigated the correlation between serum E2, salivary E2 and measurements of follicles by two-dimensional ultrasound. A previous study by Dielen et al. (2017) showed a good clinical correlation between serum and salivary E2. This pilot study provides evidence to support the hypothesis that salivary E2 could be used as a substitute for serum E2. We wanted to confirm these previous results and also investigate if there is a correlation between salivary E2 and measurements of growing follicles by ultrasound.

## Materials and methods

### Patient selection

This prospective study was part of an international, multicentre clinical trial on the replacement of serum monitoring by salivary hormone measurements (Sakkas et al., 2018). This sub-study was performed at the Department of Reproductive Medicine of Ghent University Hospital (Belgium). It was approved by the local ethics committee (reference: EC/2016/1303). Between November 2016 and January 2017 a consecutive sample of 40 patients was selected. All patients started with an IVF or ICSI attempt for various reasons. A minimum age of 21 years and maximum of 42 years were used as cut-off for inclusion. Oocyte donation treatments were excluded. Fertility problems were not otherwise specified.

### Procedure

The traditional follow-up of COS comprises assessment using vaginal sonography of follicle count and size and measurement of serum E2. All patients had an initial ultrasound on day 3 of the menstrual cycle to rule out cysts or persistent follicles before starting stimulation. During the stimulation, several visits took place according to clinician preference. The number of measurements per patient ranged between two and seven. The study added salivary sampling each time the routine serum sampling and sonography were performed. This was continued until administration of the hCG trigger (5000IU) (Pregnyl®, Merck Sharp & Dohme, Ltd., Hoddesdon, UK). HCG was administered when > 50% of the follicles reached a diameter of ≥18 millimetre.

All patients were stimulated following a short agonist scheme. Several drugs were used for stimulation. Menopur® (HP-HMG, Ferring Pharmaceuticals A/S, Copenhagen, Denmark), was used by thirty-three patients; six patients used Bemfola® (follitropin alfa, Finox AG, Burgdorf, Switzerland) and one used Gonal-F® (follitropin alfa, Serono, Geneva, Switzerland).

Phlebotomy was performed for blood sampling. Blood samples were analysed in the laboratory of Ghent University Hospital. Seventeen-beta E2 was routinely determined by immunoassay on an E170 Modular (E2 Gen III, Roche Diagnostics, Germany). Saliva was collected using the drooling technique. This technique consists of letting the saliva drip in a 2 ml tube. All patients received oral and written instructions. Saliva samples were frozen at -20° C and transported and analysed in batch at the Boston IVF Fertility Clinic using the Salimetrics E2 immunoassay according to the protocol provided by Salimetrics (Carlsbad, CA, USA).

In general, the saliva is frozen even when a sample is collected onsite for immediate assay. After thawing, any impurities are noted (e.g. food particles, discoloration) and the sample is homogenized by thorough mixing. The saliva is centrifuged and the supernatant is retained. Aliquots of the retained supernatant are assayed for E2 level. No particularly foul smell was noted here, which means nothing different than the standard odor of saliva. No extraction step is performed. The assay is a competitive-binding immunoassay. The analyte is co-incubated with a known concentration of enzyme-linked E2 (conjugated to horseradish peroxidase). After incubation, the plate is washed and incubated with tetramethylbenzidine. The resulting color is measured as Optical Density using a plate reader set to read at 450 nm.

The reported validation of the assay (per Salimetrics’ published protocol) shows a sensitivity as low as 0.1 pg/mL and a correlation of 0.8 to serum values. Moreover, inter- and intra-assay precision shows less than 9% coefficient of variation (CV) among both high and low concentrations.

The samples were run over six different days and a high, medium and low reference concentration included each run. The assay precision for the high, medium and low values was 1.8%, 4.2% and 7.3%, respectively.

The following equipment was used: Biotek ELx50 plate washer, Biotek EL 800-plate reader, Allegra X-30 centrifuge, Vortex Genie 2.

All sonographies were performed by one of three physicians in order to minimize the intra- and interobserver variability. The number of follicles, the two-dimensional size and the thickness of the endometrial lining were measured using a vaginal probe (Voluson S6, GE Ultrasound Korea, Gyeonggi-do, Korea). Follicles >10 millimetre were taken into account. In the analysis, the measurements of left and right ovary were combined since the total response of ovarian tissue is of importance and not the individual response of each ovary separately. As an outcome value, the product of the number of follicles with the mean diameter of the follicles in millimetres was calculated. This value is independent of the number of follicles, it is an indicator of how the ovarian tissue responds to the stimulation.

The values were considered in relation to the day of ovarian pick-up. Patients did not undergo an ultrasound or serum and saliva collection every day. This means that the values of each measurement point are the means of the patients who obtained investigations on that day.

### Statistics

Statistical analysis was performed by the Department of Statistics of Ghent University. The linear mixed-effects models (MIXED) procedure in SPSS Statistics 24 was used for statistical analysis.

## Results

Table I shows the demographical characteristics of the patients in this study. There was one smoker. The mean alcohol consumption was 1.87 units per week. The number of previous IVF/ICSI attempts varied between 0 and 4. For 37.5% of patients this was the first attempt.

**Table I:** Demographic characteristics of patients enrolled.

	N, Valid	Mean	Minimum	Maximum
Age (years)	40	34.4	25	42
AMH (μg/L)	40	2.09	.053	6.89
BMI	37	24.9	18	35
Starting Dose gonadotrophins or recombinant FSH (IU)	40	240.39	75	300
Total Dose (IU)	40	3146.25	975	7125

All 40 patients had basal levels of E2 on the initial ultrasound and showed no follicles larger than ten millimetres. Of the women included, 24 had an ICSI attempt, 7 an IVF attempt. Of the remaining 9 patients, 2 had an oocyte aspiration for cryopreservation of oocytes, 5 patients had insufficient ovarian response and had to undergo an escape intra-uterine insemination and the remaining 2 patients had abnormal oocytes after oocyte pick up. Nine of the 38 patients had a positive hCG test. Six patients had a clinical pregnancy (foetal heartbeat at 7 weeks pregnancy).

**Figure 1:**
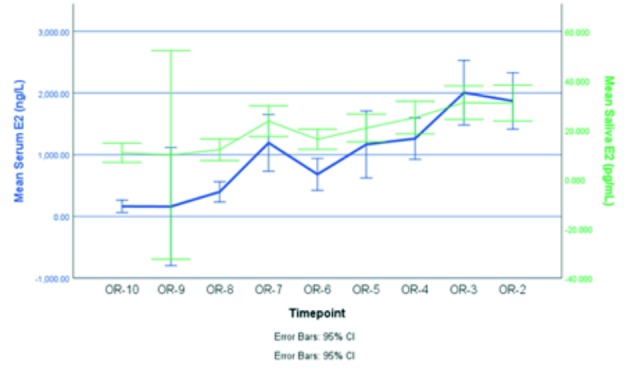
— Superposition of mean serum E2 and mean saliva E2. Mean serum E2 (blue, in ng/L) and mean saliva E2 (green, in pg/mL) for all patients with an assessment on that day are both shown on the Y-axis, with confidence interval. The X-axis indicates the time of investigation (OR = oocyte retrieval, n = number of days preceding OR) (number of observations at different timepoints: OR-10=6, OR-9=2, OR-8=7, OR-7=7, OR-6=9, OR-5=13, OR-4=17, OR-3= 18, OR-2=22).

Values of serum E2 and salivary E2 show a similar increase during stimulation (Figure 1).

**Figure 2:**
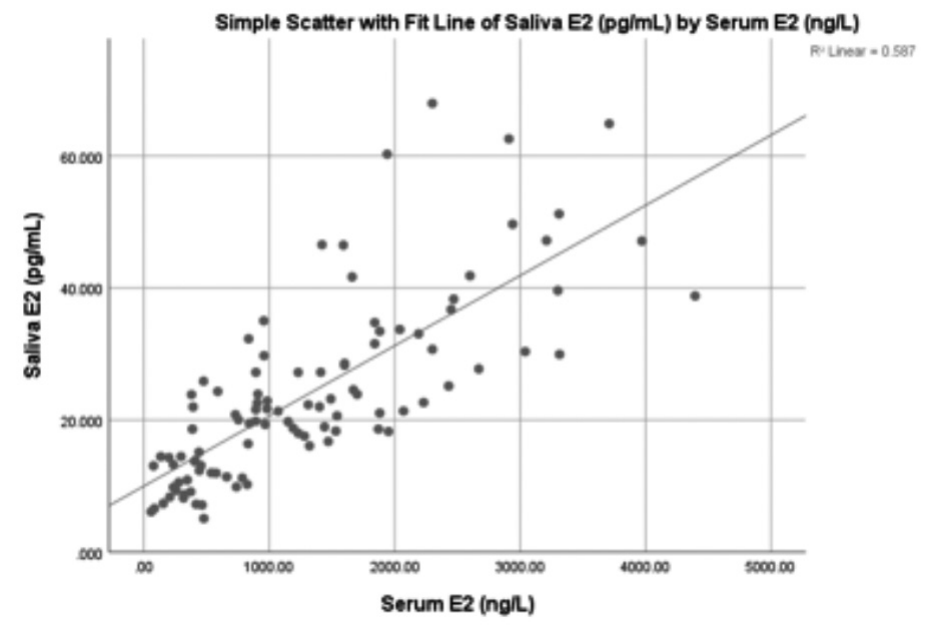
— Scatter plot showing the correlation between levels of serum and saliva E2.

A scatterplot shows a good correlation (R=0.77) between serum and saliva E2 (Figure 2). A linear mixed model shows that for every unit increase in serum E2 (+ 1 ng/L= 1pg/ml) the estimated saliva E2 concentration is expected to increase with 0.011 pg / mL [95% CI from 0.009 to 0.01, p < 0.001], which is a significant result.

Moreover, the product of the mean follicle diameter with follicle count, taken as a quantitative sonographic marker increases in relation to the time of stimulation.

**Figure 3:**
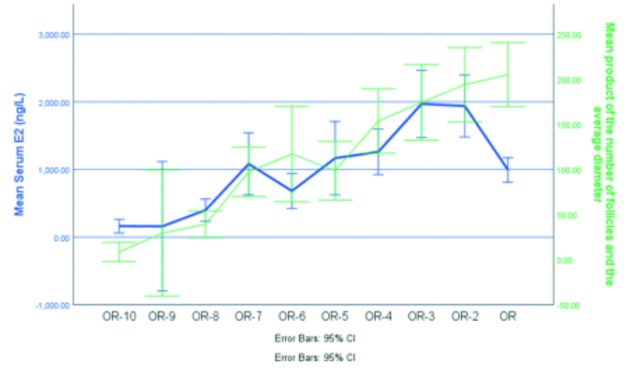
— The Y-axis shows the mean serum E2 for all patients who had an investigation on that day and the measurements of the follicles, expressed by the mean product of the number of follicles and their average diameter. The X-axis shows the time of investigation.

**Figure 4:**
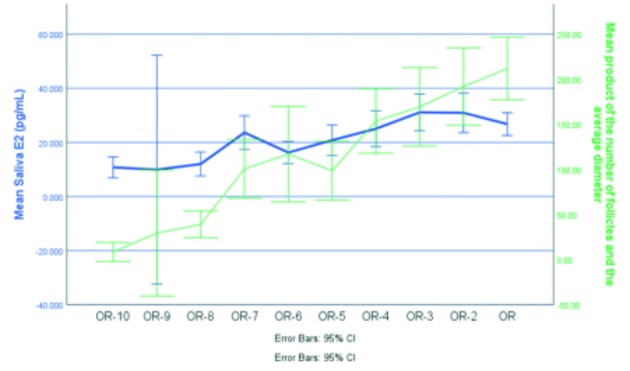
— The Y-axis shows the mean salivary E2 for all patients who had an investigation on that day and the measurements of the follicles, expressed by the mean product of the number of follicles and their average diameter. The X-axis shows the time of investigation.

Figure 3 and 4 show the comparison of levels of E2, measured either in blood or in saliva, and the measurement of the follicles by sonography, expressed as the product of the number of follicles and their average diameter. There is a similar correlation between salivary E2 and follicular dimensions, and serum E2 and follicular dimensions. When a linear mixed model with heterogeneous autoregressive covariance is performed, it appears that for every unit increase in follicular diameter (+1 millimeter) the estimated serum E2 concentration is expected to increase with 8.32 ng / L [95 % CI from 7.10 to 9.54, p < 0.001]. For every unit increase in follicular diameter (+ 1 millimeter) the estimated saliva E2 concentration is expected to increase with 0.11 pg / mL [95 % CI from 0.09 to 0.13, p < 0.001].

## Discussion

Monitoring of ovarian stimulation for IVF/ICSI is routinely performed using serum sampling for E2 and sonographic measurements of follicles. Some studies claim that ultrasonography alone is enough. A meta-analysis concluded that monitoring COS by ultrasonography alone is unlikely to cause substantial reduction in the number of oocytes retrieved (moderate quality evidence) or alter the chance of achieving a clinical pregnancy (low quality evidence) (Martins et al., 2014). A Cochrane review found similar results and concluded that there is no firm evidence that stimulation monitoring using TVUS alone is less effective or less efficacious than combined monitoring using TVUS and E2 assays, both with regard to clinical pregnancy rates and the incidence of OHSS. However, the overall quality of the included studies was low. The review concluded that a combined monitoring protocol including both TVUS and E2 assay is seen as precautionary good clinical practice and as a confirmatory test in a subset of women to identify those at high risk of OHSS (Kwan et al., 2014).

Because of the burden for the patient of taking blood samples for E2 assessments, a less invasive alternative would be welcomed. Boston IVF introduced the first needle-free saliva test. Patients can collect their saliva at home in less than five minutes and drop off their samples for analysis each morning. Saliva testing has numerous advantages. It is non-invasive, simple to perform, safe for the patient and practitioner, stress free, painless, private and convenient for the patient and his or her physician (Kells et al., 2009). Moreover, E2 in serum is strongly bound to sex hormone globulin (SHBG) and weakly to albumin. In women, it is assumed that only about 1% of E2 is actually present as free hormone in blood. Only a small free fraction functions as the active hormone and can be measured by reference methods such equilibrium dialysis (ED) coupled to liquid chromatography with tandem mass spectrometry (LC-MS/MS) in serum. In saliva, hormones are largely present in their free, unbound form, which suggests that measuring the free fraction of E2 in saliva could be a more natural method than performing measurements in serum (Fiers et al., 2017).

In the present study, we found a good correlation between serum E2 and the product of the mean diameters of the follicles. This is similar to the results of previous studies. In normal menstrual cycles there is a good correlation between the diameter of the dominant follicle and the serum E2 concentration (Bakos et al., 1994; DeCherney et al., 1984). Looking at stimulated IVF cycles with clomiphene citrate, mean E2 concentration increases in a linear way as well as mean follicular diameter of the dominant follicle. Vargyas et al. (1982) found a significant correlation between mean follicular diameters and E2 levels (r=0.981, p<0.001). A significant correlation between total follicular volume and mean E2 concentration was also demonstrated (r=0.978). In this study group, each follicle that was 18 millimetre in diameter generated an average of 460 pg/ml of E2 concentration in serum. Hull et al. (1986) concluded that total follicular volume of both ovaries and the total follicular volume of the ovary containing the dominant follicle are positively correlated with preovulatory serum E2 levels. The size or volume of the largest follicle was not related to serum estrogen levels. This can be explained by the fact that the largest follicle is not necessarily the most estrogenic.

We found a good correlation between salivary and serum E2 as well. The levels of E2 in serum and saliva increase in a day-to-day manner from the start of stimulation until day -2 of oocyte retrieval. A high level of serum E2 is associated with a high level of saliva E2. Celec et al. (2009) found a correlation between salivary and plasma levels in the menstrual cycle. The curves of salivary E2 are similar to curves reported in other studies for E2 in plasma. Our findings are similar to previous findings (Dielen et al., 2017; Sakkas et al., 2018; Zimon et al., 2013). Sakkas et al. (2018) found a good correlation between saliva and serum E2, with a correlation coefficient of 0.88. This study, as well as the study by Dielen et al. (2017) concluded that saliva can be a good surrogate for free E2 in women undergoing COS. Patient survey results showed that saliva sampling was the preferred method of analysis, associated with less anxiety and more likely to be recommended to friends (Zimon et al., 2013). However, salivary estradiol testing is not yet routinely commercially available and further clinical testing is necessary to find out if measuring of E2 in saliva is reliable in routine clinical practice. In another study, plasma and salivary E2 concentrations were compared with ultrasound in cycles of ovulation induction with human menopausal gonadotrophin (Hardiman et al., 1990). In this study, serum E2 correlated poorly with the number of mature follicles but a highly significant correlation between serum and salivary E2 was shown on day 7. Therefore, it is no surprise that salivary E2 did not correlate with the number of mature follicles (>18 millimeter) on day 7. The authors found a weak correlation between salivary E2 and the number of follicles measuring 16 millimetres or more (r = 0.30). They concluded that TVUS is the most accurate method of monitoring responses to human menopausal gonadotrophin and that the estrogen assay (in serum or saliva) provides no additional useful information. In our study, performed with IVF/ICSI attempts, both serum and saliva E2 correlated with the total number of follicles >10 millimetre. We conclude that estrogen levels in serum and saliva correlate with the total number of developing follicles. During stimulation, there are a lot of follicles that develop in an asynchronous manner. Not all of the follicles become mature. So, E2 in saliva and serum correlates with the total number of developing follicles rather than the number of mature follicles observed on ultrasound. Serum E2, as well as saliva E2, correlates with sonographically measured follicle growth in the same manner. We therefore suggest that saliva E2 can be a good alternative for serum E2 in monitoring IVF/ICSI cycles. This could simplify the follow-up of COS and making it more patient friendly.

However, measuring E2 in saliva, induces a set of problems both in terms of matrix complexities and concentration. As observed, only 1% of the serum total E2 value is present in saliva. This low concentration makes the investigation of E2 in saliva technically complex. Another limitation of saliva resides in the pre-analytic sample quality. Spitting has to be avoided to avoid clots. Blood contamination due to teeth brushing, particular food or beverages can make the sample unusable. E2 values are elevated in salivary samples with blood contamination. This can adversely influence clinical interpretations. Elevated salivary E2 results should be treated with caution (Evans et al., 1980; Collins et al., 1981; Berthonneau et al., 1989; Fiers et al., 2017). If there is discordance with sonography findings, serum E2 may need to be analysed to confirm.

The big advantage of saliva is that it can be produced at home. In rural areas, in our stressful, busy lives, the patient can produce saliva in her own free time and put it in a post bus or use a courier delivery service, which is frequently used in modern countries (Zimon et al., 2013). Salivary sampling should be seen in a bigger picture of patient-friendly IVF monitoring. Several studies have reported good clinical outcomes with sonographic monitoring at home. The combination of home sonography monitoring with salivary E2 sampling could simplify the monitoring of ovulation stimulation (Gerris & De Sutter, 2010; Gerris et al., 2016) and remove any doubts related to hyper- or hypo- response.

A further benefit of less invasive diagnostics or home testing is the possible limit in stress experienced by the patient. Multiple venepunctures experienced during monitoring can add to stress levels that may impact outcome or the possibility of returning for a subsequent cycle (Rooney and Domar, 2016).

## Conclusion

The most optimal method of monitoring ovarian stimulation in IVF/ICSI cycles remains debated. The 2014 Cochrane study concludes that the combination of ultrasound and E2 may need to be retained as precautionary way of good clinical practice and as a confirmatory test for those at risk for OHSS.

In our study, a strong correlation between saliva and serum E2 was found. Moreover, both seem to correlate with the product of the number of follicles and the average diameter, measured with sonography. We conclude that if E2 is used for monitoring of ovarian stimulation, salivary E2 seems to be at least a viable alternative for serum E2. This creates an opportunity for simplification of monitoring IVF/ICSI cycles.
